# The Awareness of Negative Pressure Pulmonary Edema in the Medical Intensive Care Unit

**DOI:** 10.7759/cureus.10251

**Published:** 2020-09-04

**Authors:** Qian Zhang, Jonathan Vayalumkal, John Ricely, Sarah Elrod, Ahmad Raza

**Affiliations:** 1 Internal Medicine, Abington Hospital-Jefferson Health, Abington, USA

**Keywords:** negative pressure pulmonary edema, nppe, noncardiogenic pulmonary edema

## Abstract

Negative pressure pulmonary edema (NPPE) is a noncardiogenic-related rapid onset of bilateral pulmonary edema secondary to various etiologies that lead to upper airway obstruction. Despite the fact that it is more commonly seen in the emergency department (ED), pediatric intensive care unit (PICU), and the postanesthesia care unit (PACU), there is a lower disease prevalence in the medical intensive care unit (MICU) as it sometimes could be overlooked upon. Prompt treatment often leads to favorable clinical outcomes. We detail a 55-year-old lady with a past medical history of nephrolithiasis, obesity, and obstructive sleep apnea presented with right flank pain due to right kidney subcapsular hematoma and left hydronephrosis due to distal ureteral calculus requiring urological intervention. She unfortunately subsequently developed NPPE requiring MICU level of care after reintubation. Successful extubation was achieved two days later as timely recognition of NPPE led to proper treatment. She was downgraded to general medicine service and discharged without complications.

## Introduction

Negative pressure pulmonary edema (NPPE) is a phenomenon that could often be life threatening in patients due to acute upper airway obstruction. It is reported that there are up to 12% of patients with NPPE despite that the actual incident could be a lot higher as clinicians are often unaware of such disease process [1]. NPPE is a form of noncardiogenic pulmonary edema that could be seen in postoperative patients following extubation [2]. The pulmonary edema arises from the negative pressure that the patient generates due to upper airway obstruction, which leads to a diffuse alveolar edema process secondary to the hydrostatic pressure [3]. Proper disease recognition is required to initiate prompt treatment to ensure patient safety. Patients often respond rapidly from mechanical ventilation and diuretics. 

## Case presentation

**Figure 1:**
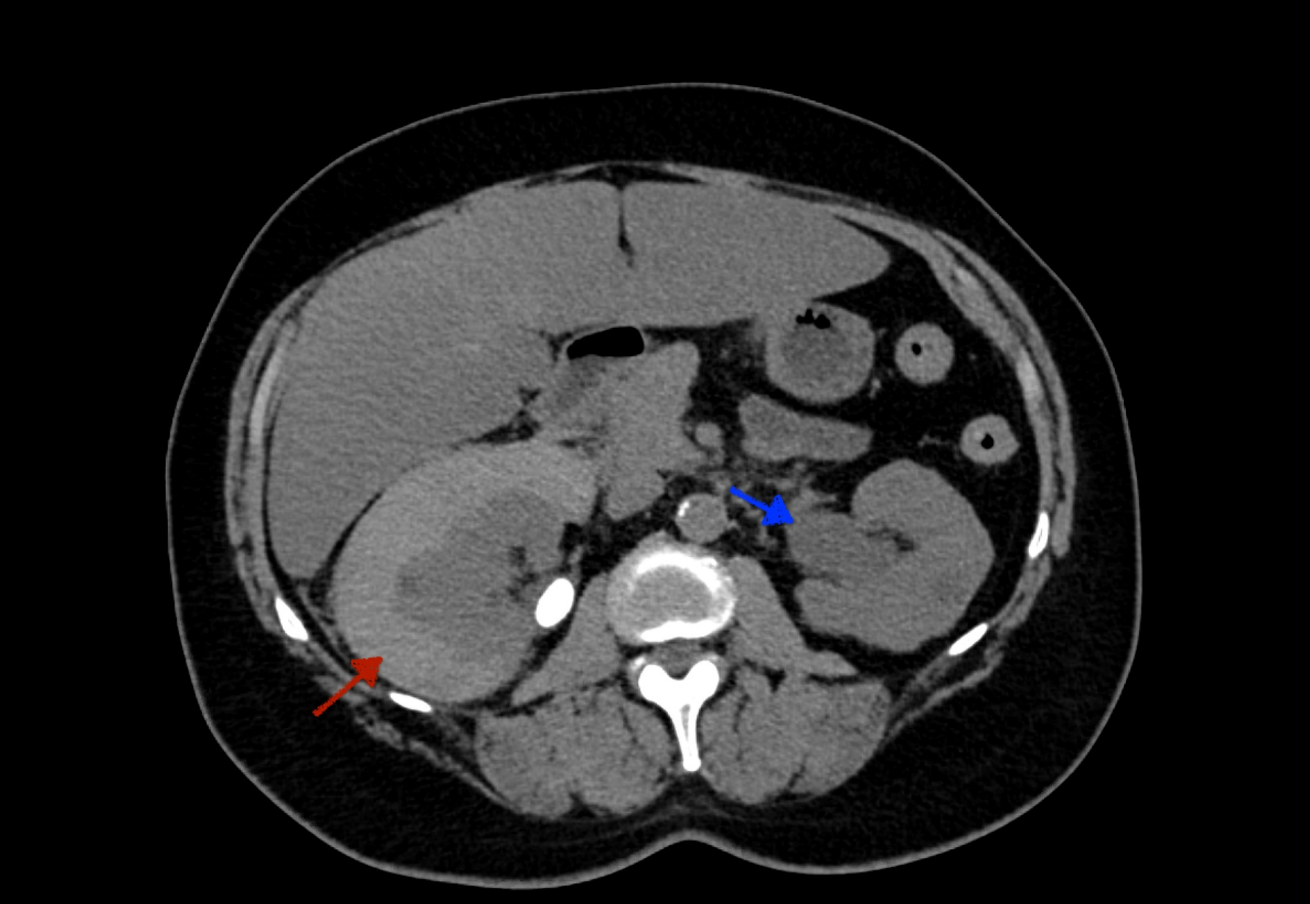
CT Scan A subcapsular hematoma of the right kidney (red arrow) and mild to moderate left hydronephrosis (blue arrow) due to distal ureteral calculus.

**Figure 2:**
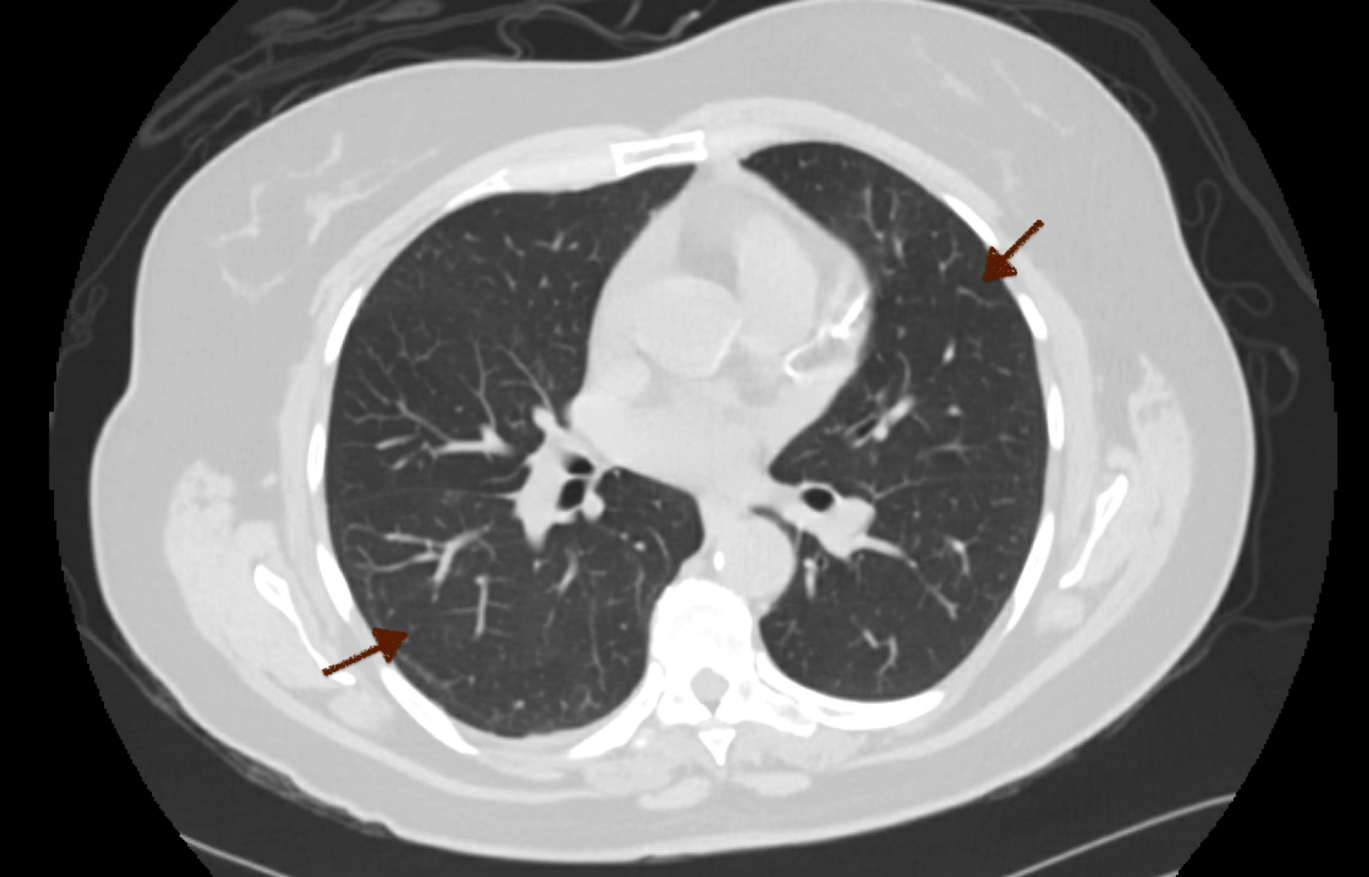
CT Scan No pulmonary opacities noted bilaterally (red arrows). No cardiomegaly appreciated.

A 55-year-old woman with a past medical history of nephrolithiasis, dermatomyositis, gastroesophageal reflux disease, and obstructive sleep apnea was admitted to the hospital with the chief complaint of right flank pain with associated nausea and vomiting. She was afebrile and hemodynamically stable with the blood pressure of 138/78 mmHg, heart rate of 96 beats per minute, and oxygen saturation of 99% on the room air. Physical examination showed normal bilateral air entry without wheezing or crackles appreciated. She had a regular rate and rhythm without murmur, rubs, and gallop noted. The patient did not appear to be volume overloaded as there were no findings of S3, jugular venous distention, or pitting edema. Serum creatinine was elevated to 1.73 mg/dL from the patient’s baseline of 0.80 mg/dL (normal range: 0.84-1.21 mg/dL). Cardiac NT-proB-type natriuretic peptide (BNP) was 25 pg/mL (normal range: <100 pg/mL). CT scan of the body discovered a subcapsular hematoma of the right kidney and mild to moderate left hydronephrosis due to distal ureteral calculus (Figure 1). No significant pulmonary findings were noted (Figure 2). She was seen and evaluated by urology with the decision to undergo a bilateral ureteroscopy with basket retrieval of left ureteral stone with stent placement.

**Figure 3:**
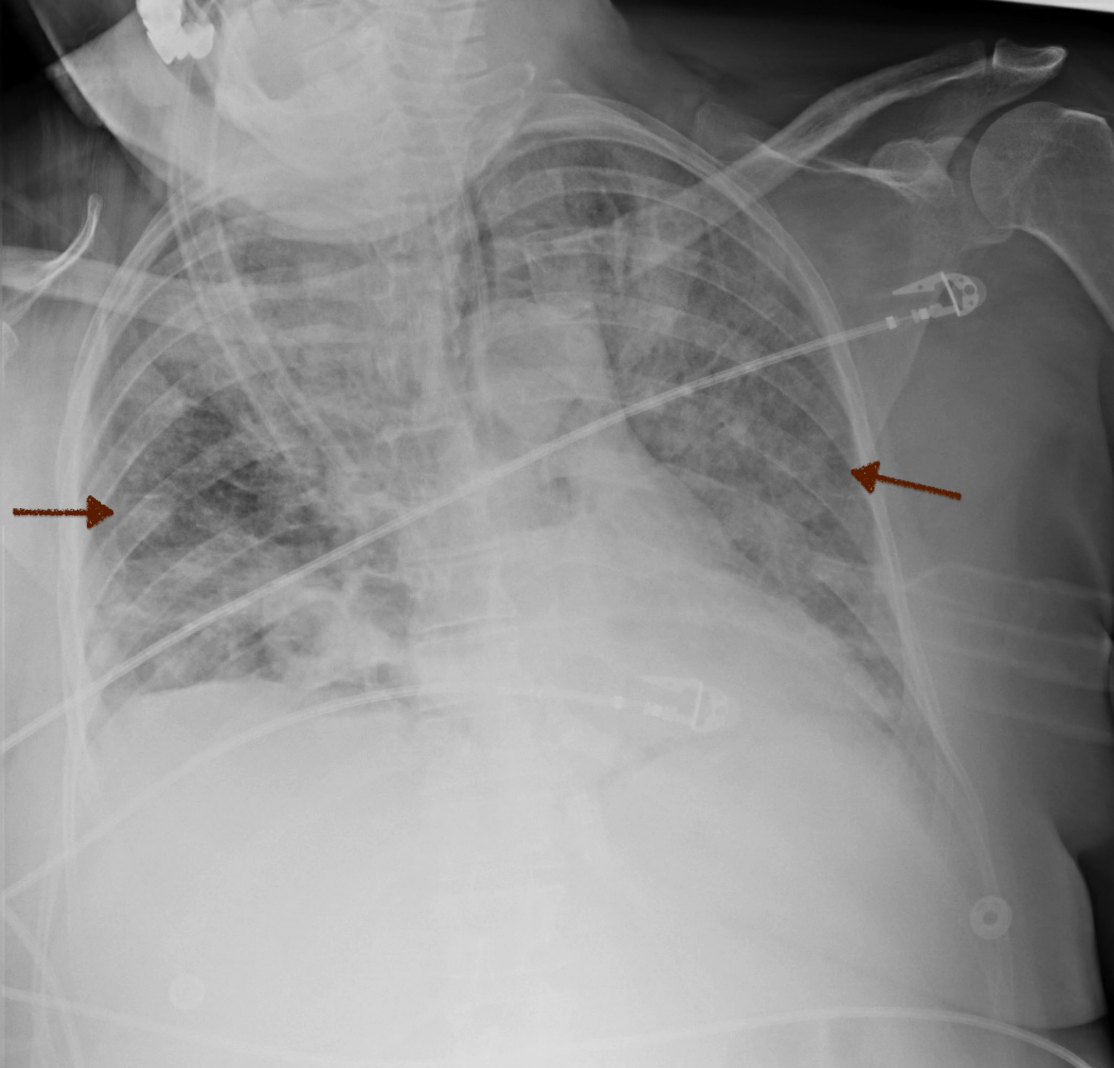
Chest X-Ray Diffuse bilateral airspace infiltrates postsurgery (red arrows).

On day 2 of hospitalization, she remained to be hemodynamically stable without respiratory distress. Furthermore, she was found to have a body mass index of 30.8 kg/m^2^ and a Mallampati III airway preoperatively. Intraoperatively, the patient was pre-oxygenated with 100% oxygen by mask for two minutes and underwent intravenous induction and easy mask ventilation without airway obstruction. She remained to be clinically stable without complications. She was subsequently transferred to the postanesthesia care unit (PACU) with adequate spontaneous respirations postextubation. However, she suddenly became restless, not following commands, and had worsened respiratory effort. Blood pressure was 188/104 mmHg, heart rate was 131 beats per minute, and respiratory rate was 38 breaths per minute. Moreover, the patient became lethargic with the pulse oximetry suddenly dropped to 74%-85%. A nonrebreather (NRB) was placed, and Narcan® 80 mcg was administered without mental status improvement. Arterial blood gas (ABG) revealed a respiratory acidosis with a pH of 7.15 (normal range: 7.35-7.45), a carbon dioxide level of 50.5 mmHg (normal range: 35-45 mmHg), an oxygen level of 46.1 mmHg (normal range: 75-100 mmHg), and a bicarbonate level of 17.8 mmol/L (normal range: 22-26 mmol/L). The patient was subsequently re-intubated as she was in severe respiratory distress. There were notable copious secretions found in the oral cavity. Postintubation chest radiograph (CXR) revealed diffuse bilateral airspace opacity that was consistent with pulmonary edema (Figure 3). The patient was upgraded to the medical intensive care unit (MICU) for the management of acute hypoxemic and hypercapnic respiratory failure. She received diuretics with the goal of maintaining a negative fluid balance.

**Figure 4:**
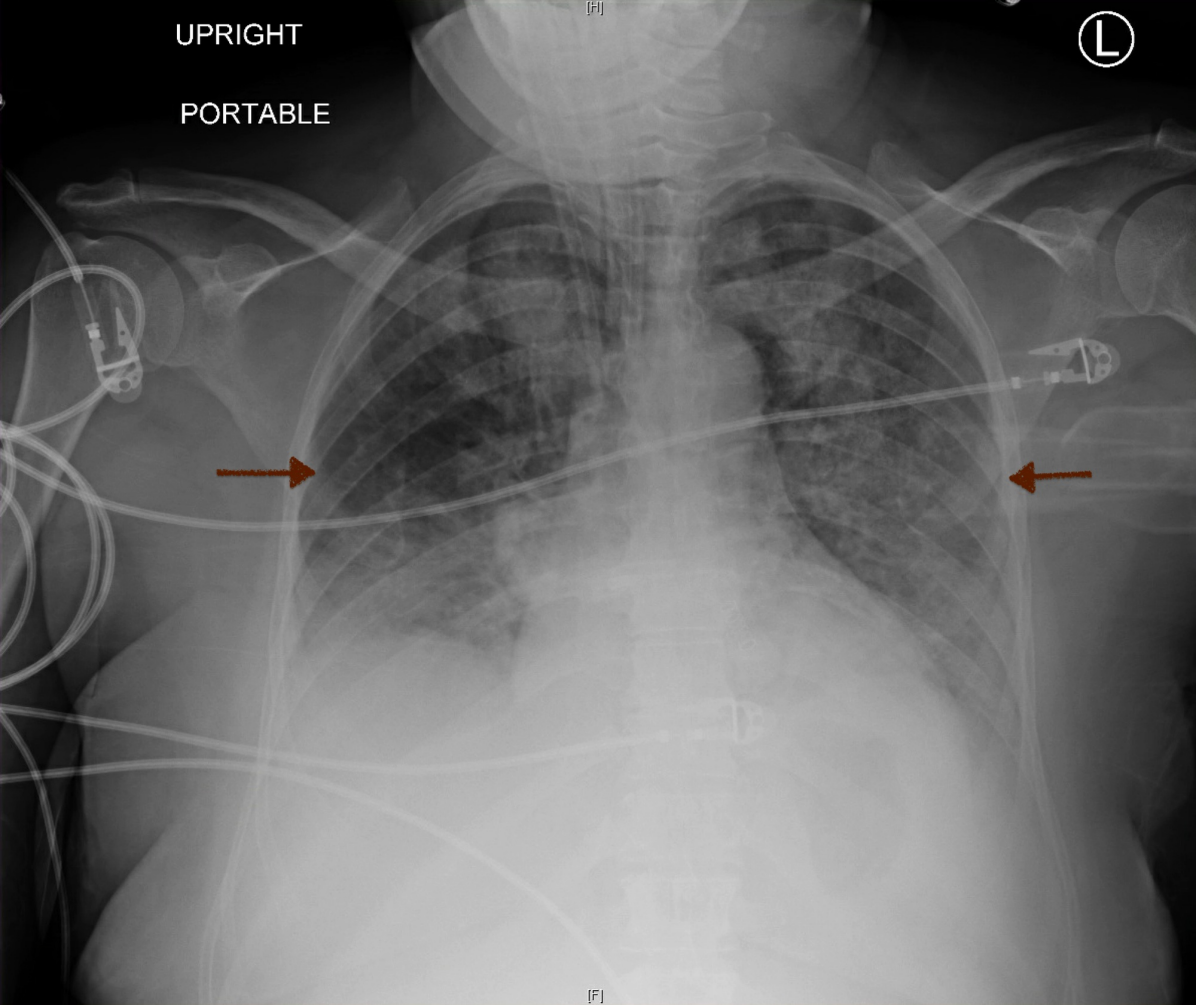
Chest X-Ray Improvement in aeration with a decrease in bilateral airspace infiltrates (red arrows). L: Left. H: Head. F: Feet.

On day 3 of hospitalization and postoperative day 1, ABG revealed pH 7.29 (normal range: 7.35-7.45) with carbon dioxide level of 30 mmHg (normal range: 35-45 mmHg), the oxygen level of 57 mmHg (normal range: 75-100 mmHg), and bicarbonate level of 18 mmol/L (normal range: 22-26 mmol/L) as the patient was on continuous mandatory ventilation (CMV) with a respiratory rate of 14 breaths per minute, tidal volume of 400 mL, 90% fraction of inspiratory oxygen (FiO_2_), and 5 cm H_2_O of positive end-expiratory pressure (PEEP) with normal peak and plateau pressures. Oxygen requirement was eventually weaned down to 50% FiO_2_ and the patient was in a net negative one liter of fluid balance with creatinine level at 2.34 mg/dL (normal range: 0.84-1.21 mg/dL). CXR showed improved aeration along with decreased bilateral airspace infiltrates (Figure 4).

**Figure 5:**
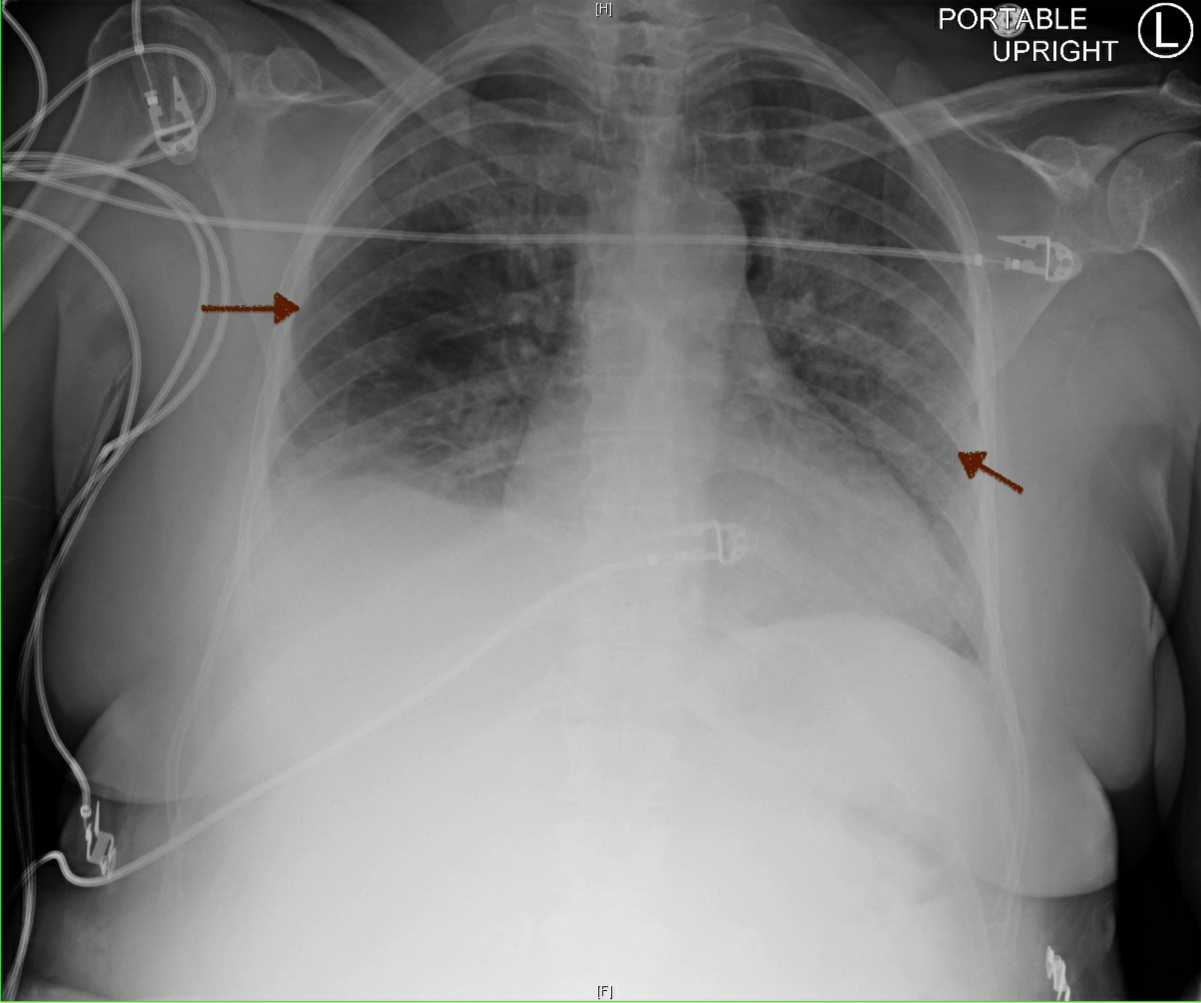
Chest X-Ray Interval extubation along with gradual improvement of airspace opacities (red arrows). L: Left. H: Head. F: Feet.

On day 4 of hospitalization and postoperative day 2, the patient was weaned down to 40% FiO_2_ and was successfully extubated. The CXR revealed marked improvements in diffuse bilateral alveolar opacity compared to the prior CXR (Figure 5). Moreover, the patient was downgraded to the general medicine floor while sating well on two liters of oxygen by nasal cannula. No wheezes, rhonchi, or crackles were appreciated on physical exam. 

**Figure 6:**
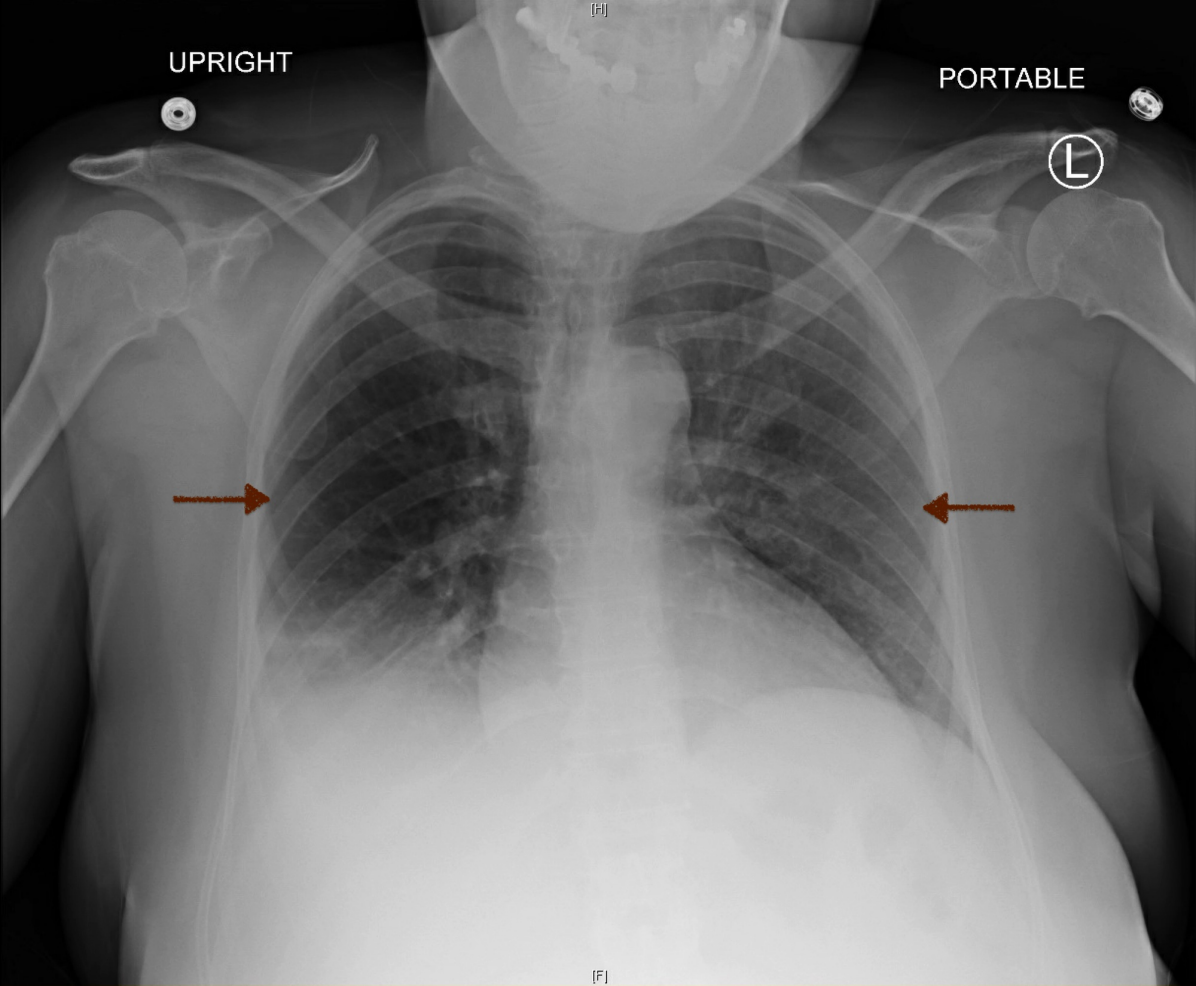
Chest X-Ray Continued improvement of the bilateral opacities (red arrows). L: Left. H: Head. F: Feet.

On days 5 to 7 of hospitalization, her oxygen requirement was weaned down to room air while the diuretic therapy was subsequently discontinued. The creatinine level improved to 1.38 mg/dL (normal range: 0.84-1.21 mg/dL). CXR revealed continued improvement of the bilateral airspace opacities comparing to the prior imaging (Figure 6). She was deemed medically clear for discharge given the marked improvement of respiratory status. 

## Discussion

NPPE occurs when there is an imbalance of pressure gradient in the alveoli of the lung that subsequently leads to the flooding of the alveolar sac. The normal physiology of the lung revolves around the filtering of the fluids from the alveolar capillaries into the interstitium where it is then deposited into the lymphatic system of the lung for clearance. This intrinsic process is driven by factors including the coefficient of the capillary permeability, the hydrostatic pressure gradient between the alveolar-capillary lumen and the alveolar interstitium, as well as the protein osmotic pressure gradient of the vasculature and interstitium. The hydrostatic pressure elevates in the setting of NPPE, which generates a fluid capacity that exceeds the clearance ability of the lymphatic system, resulting in the abnormal accumulation of fluids in the interstitium and alveolus. These changes would be present on the CXR as a new onset of flash pulmonary edema with bilateral alveolar infiltrates [3]. This phenomenon ultimately affects the normal gas-exchange process in the alveoli which results in acute respiratory distress. 

Furthermore, the elevated hydrostatic pressure noted in NPPE is due to the increase in intrathoracic negative pressure against an obstructed upper airway [4]. The common causes of NPPE include laryngospasm, upper airway infection, and malignancy [3,4]. Moreover, NPPE is mostly seen in the emergency department (ED), pediatric intensive care unit (PICU), and the PACU. However, the incident of NPPE in the MICU is extremely low per a retrospective research study conducted by Koh et al. [5]. Only five patients were diagnosed with NPPE secondary to the acute epiglottis, right bronchus intermedius stenosis status post stent, goitrous obstruction, and strangulation in a five-year period of a 1,500-bedded tertiary care hospital. Despite the rarity of NPPE in the MICU setting, it is important to acknowledge that a broad range of etiologies encountered in the MICU including but not limited to hypothyroidism, difficult intubation, Ludwig angina, and severe patient-ventilator asynchrony may lead to the development of NPPE [3]. Extra attention should also be paid in mechanically ventilated patients with high body mass index (BMI) in the setting of untreated obstructive sleep apnea (OSA) as they are at potential risk of developing NPPE [6]. 

However, it is essential to differentiate the cause of the pulmonary edema between cardiogenic and noncardiogenic by using the proper clinical context. It is sometimes difficult to differentiate as the upper airway obstruction may be more subtle in the certain patient population, which makes the connection between the obstruction and the subsequent pulmonary edema less obvious. This hardship is further complicated as physicians may be unaware of the existence of such a clinical entity that could further impact the treatment. In a retrospective research analysis by Kollef and Pluss, there appeared to be a positive correlation between the number of risk factors of noncardiogenic pulmonary edema and the severity of NPPE [7]. 

Timely treatment of NPPE is essential after the recognition of NPPE. The therapy revolves around urgent endotracheal intubation with positive-pressure ventilation. In patients who developed NPPE due to prolonged biting or mechanical obstruction of the endotracheal tube, uptitrating the sedation or even the use of paralytics could be considered to alleviate the upper airway obstruction [3]. Most patients respond rapidly to the proper treatment as per a retrospective review conducted by Fremont et al. [8]. Out of 341 patients, 10 were discovered to have acute pulmonary edema with the postobstructive mechanism. The average rate of alveolar fluid clearance over an eight-hour span postintubation was 14.0 ± 17.4% per hour. In addition, other treatment options are similar to that of acute respiratory distress syndrome despite a lack of current research study as supporting evidence. It includes the use of low tidal volume ventilation to decrease the likelihood of ventilator-associated lung injury as well as diuretics if the patient appears to be volume overloaded along with a lack of contraindications such as acute kidney injury or shock [3]. 

Our patient had a sudden onset of acute hypoxic hypercarbia respiratory failure right after the initial extubation. There were multiple risk factors that could be contributory to the NPPE, including her obese body habitus with underlying OSA, Mallampati III airway, and copious secretions noted in the oral cavity. Her pulmonary edema was likely noncardiogenic given the lack of history of cardiovascular disease, normal cardiac BNP, as well as a lack of physical evidence to suggest an initial volume overloaded status. It is also important to note the lack of active pre-existing pulmonary disease seen on the initial CT imaging. Her condition improved rapidly as evidenced by the serial of CXR findings post reintubation with positive ventilatory support along with daily diuresis. 

## Conclusions

There should be an increase in awareness of NPPE especially among healthcare providers working in the MICU as it could often be overlooked due to a relatively lower disease incidence along with lack of recognition. It is important to differentiate this acute onset of pulmonary edema from cardiogenic etiologies by evaluating the patient holistically in the proper clinical setting. Timely treatment in patients with reversible causes of upper airway obstruction often leads to favorable outcomes. Patient safety starts with timely and proper awareness. 
